# Periodic lateralized epileptiform discharges as manifestation of pneumococcal meningoencephalitis

**DOI:** 10.1186/1755-7682-4-23

**Published:** 2011-06-25

**Authors:** Francisco Hernández-Fernández, Eva Fernández-Díaz, Jose M Pardal-Fernández, Tomás Segura, Jorge García-García

**Affiliations:** 1 Department of Neurology. Complejo Hospitalario Universitario de Albacete (Hermanos Falcó,37). Albacete (Postal code: 02/2006). Spain; 2 Unit of Neurophysiology. Complejo Hospitalario Universitario de Albacete. Albacete. Spain

## Abstract

Periodic Lateralized Epileptiform Discharges (PLEDs) are usually seen in the context of destructive structural lesions of the cortex, more frequently in acute ischemic stroke and less common in tumours and meningoencephalitis, specially herpes simplex virus. Its origin and prognosis are uncertain but it is known that PLEDs are linked to epilectic seizures, including status epilepticus.

We report on a 75-year old woman with pneumococcal meningoencephalitis who presented altered level of consciousness, acute focal deficits, convulsive seizures and PLEDs in left hemisphere. The finding of PLEDs on the electroencephalogram is related to focal lesions of heterogeneous origin, which up to date, have not been documented in pneumococcal infections of the central nervous system. Our case highlights the importance of identifying and addressing any modifiable etiologic factors of PLEDs.

## Introduction

Electroencephalogram (EEG) in acute meningoencephalitis is always abnormal. In most cases of nervous central system infectious (CNS) diseases a diffuse slowing is visible with bilateral paroxystic and synchrone affectation [1]. One of the most frequent paroxystic complexes is the pattern of periodic lateralized epileptic form discharges (PLEDs). This is defined by presence of a pattern of repetitive paroxysmal slow or sharp waves, uni or bilateral at intervals of between 0.5 to 3 seconds [2,3]. The morphology of PLED is epileptiform. However, due to some of their characteristics they are considered as interictal pattern. In any case, it is usually associated with epileptic seizures [4], even status epilepticus, which must be excluded and treated appropriately.

There is a wide variety of potential PLEDs etiologies, most of them focal lesions, of which acute ischemic stroke is the most frequent cause in all series. Although PLEDs may also appear in tumours, haemorrhages or CNS infections, this waveform is considered a quite specific EEG pattern for herpes simplex virus encephalitis [5], but they are also related to inflammatory processes of different origins such as neurosyphillis [6], demyelinizing diseases [7], neurocysticercosis [8], influenza [9], neuro-Behcet's disease [10] or bacterial meningoencephalitis, including Q fever [11].

We present a patient with pneumococcal meningoencephalitis associated with PLEDs on the EEG in the symptomatic hemisphere. This case exemplifies the importance of identifying any modifiable etiologic factors of PLEDs.

## Case Report

A 75-year-old woman with a history of high blood pressure, anticoagulation for chronic auricular fibrillation and multiple endocrine neoplasia (type 1 MEN), was on chronic treatment with furosemide and acenocumarol. About eight years earlier she suffered subtotal parathyroidectomy and surgery for pituitary macroadenoma, with subsequent local radiotherapy. She had not been vaccinated against pneumococcus.

She was taken to hospital by her family after finding her unconscious in her home. During her stay in the emergency department, she had a focal seizure with secondary generalized tonic clonic convulsion treated with 10 mg of diazepam. This seizure lasted no longer than one minute. In the first assessment performed by the neurologist after the seizure, the patient was unconscious, without response to verbal stimuli, did not open eyes on pain nor localising painful stimuli on the right hemibody. Given the suspicion of acute ischemic stroke in the left middle cerebral artery with early-onset seizures a cranial computed tomography (CCT) scan was them performed, showing postsurgical changes in the left temporal region and pituitary gland without signs of acute ischemia or focal lesions. A transcranial and cervical duplex ultrasound study was them performed showing no signs of significant cervical stenosis or relevant asymmetry in the intracranial arteries. Analysis showed INR of infra therapeutic range (1.34), leucocytosis of 18,320/mm^3 ^with neutrophilia of 93%. In biochemistry, the only abnormal value was a creatinine of 1.3 mg/dl. During the following hours she developed fever although her level of consciousness improved. She was drowsy, with global aphasia and right hemiplegia with spontaneous movements in the left hemibody. Thoracic radiography showed right alveolar infiltration. Electrocardiography showed atrial fibrillation of 95 beats per minute. With the suspected diagnosis of cardioembolic stroke and aspiration pneumonia the patient received antithrombotic treatment with 300 mg of aspirin and empiric antibiotic treatment was started with amoxicillin-clavulanic acid at doses of 2 g IV three times a day (tid).

**Figure 1:**
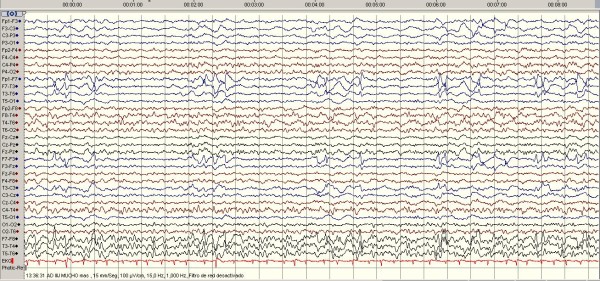
**The EEG on day 2 showed periodic lateralized epileptiform discharges (PLEDs) every 3-4 seconds in the left frontal and temporal lobes, and theta slowing in right hemisphere**.

The following morning the patient was still drowsy with right hemiplegia and aphasia. Suddenly she began to suffer myoclonic jerks in her right hemibody. These repetitive movements lasted only seconds. Nevertheless, suspicion of non-convulsive status epilepticus raised. An emergent EEG (Figure 1) was them performed. The test showed interhemispheric asymmetry defined by: 1) Left hemisphere: Slow, irregular, non-reactive delta activity 3-4 Hz, low voltage, 20-30 mcV. The recording was interrupted by appareance of large-amplitude paroxysmal abnormalities (150-200 mcV) in temporal region, presenting every 3-4 seconds. The duration of the paroxysms was 1-2 seconds. There was no evidence of superimposed fast activity, all findings deemed suggestive of PLEDs. No distortion of the contralateral hemisphere was seen, 2) Right hemisphere: slow irregular persistent theta rhythm, 5-6 Hz, of around 60-80 mcV amplitude, without structure or clear reactivity to stimuli. These findings were interpreted as non-specific encephalopathy with signs of left hemispheric lesions, which in her clinical context, pointed to acute ischemic origin. However, a further CCT did not show areas of ischemia. The possibility of herpes simplex virus encephalitis was then suspected, a disease where PLEDs have also been described frequently. On lumbar puncture a liquid with a purulent appearance was drawn, with 2,510 leukocytes/mm3 (95% polymorphonuclear), 2.89 mg/dl proteins, 16 mg/dl glycorrhachia. Abundant polymorphonuclear leucocytes and encapsulated gram-positive cocci were observed in Gram stain, and finally, detection of pneumococcal capsular antigen was positive.

**Figure 2:**
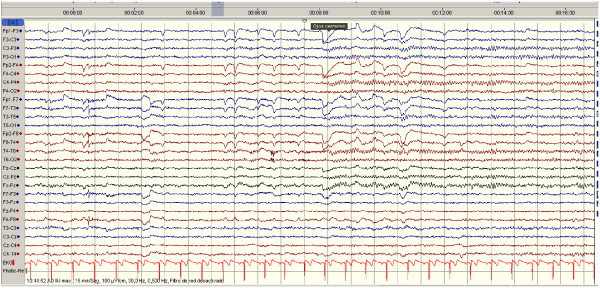
**The EEG after treatment with antibiotics reported as normal showing no PLEDs**.

Treatment was started with meropenem 1 g tid, vancomycin 1 g tid, dexamethasone 4 mg tid and phenytoin 100 mg tid. The patient was admitted to ICU, requiring sedation, orotracheal intubation, mechanical ventilation and noradrenaline perfusion during the first 48 hours. After withdrawing sedation, extubation was possible at nine days. The fever and leukocytosis disappeared and the CSF normalised (14 leucocytes 6 days after the first lumbar puncture). The response to treatment was dramatic with good recovery. Given that the diagnosis was confirmed microbiologically and clinical outcome was favourable, further neuroimaging studies were not performed. She was stable on release with a good level of consciousness, adequate orientation and no focal neurological disorders. A second EEG was performed showing no PLEDS or others paroxystic complexes (Figure 2).

## Discussion

We present a patient with diagnosis of pneumococcal meningoencephalitis, presenting focal neurological deficits in the first hours of the process, epileptic seizures and PLEDs on the EEG. The initial clinical expression and EEG were compatible with an acute ischemic stroke which led to a missed diagnosis at that moment. To the best of our knowledge, up to now no direct relationship has been established between pneumococcal infection and PLEDs.

The first description of lateralized paroxystic activities is attributed to Alajouanine et al [12], although the term was first suggested by Chatrian [13] in 1964. PLEDs are defined as complex acute periodic foci located in a hemispheric area, which repeat every 1-3 seconds, sometimes with ability to spread, followed by slow waves. They have been related to epileptic seizures in a high percentage [14], most of them focal motor seizures with or without secondary generalization, but also with focal and generalized status. The seizures typically appear in the first days, mainly during initial 24 hours.

The presence of seizure is a clinical expression of neuronal damage, for example acute ischemia, and it is associated with metabolic changes. The origin of PLEDs is obscure, but they could be related with hyperexcitability of neuronal populations of the cortex. After massive depolarisation, neurons are repeatedly excited by unknown afferent inputs, triggering the typical periodic pattern of depolarisation and refractory period seen on EEG [3]. The PLEDs are not modified by stimuli or sleep. They usually appear in the first days after the lesion, to be gradually replaced by slow, irregular focal activity. It is necessary to distinguish between PLEDs, with simple configuration and uniform discharge, and PLEDs-plus [15], characteristically accompanied by a high frequency, low amplitude discharge rhythm and with a trend to be associated with partial seizures. These abnormalities were not seen in our recording. There is no standard management for the diagnosis, prevention and treatment of seizures associated to PLEDs although it would be logical to use intravenous anticonvulsant drugs capable to reach blood levels quickly, and with a good side effect and interaction profile [16].

PLEDs are not a specific pattern of neurological diseases only are a support in the diagnosis process. The presence of PLED in patients with focal deficits and seizures is usually suggestive of stroke. Nevertheless this case illustrates the important of identifying treatable and modifiable etiologic factors of PLEDs. The neurological findings in our patient, particularly aphasia and hemiparesis, showed a better correlation with left hemispheric slowing and PLEDs in EEG, while the neuroimaging remained normal. These data suggest a functional origin and not permanent organ damage like a large infarct. The correct diagnosis and treatment resulted in complete recovery. So when investigating PLEDs etiology, pneumococcal encephalitis as a cause must not be forgotten.

*Streptococcus pneumonia *is the most common cause agent of bacterial meningitis in adults over 50 years-old. It is known that pneumococcal meningitis involves a greater degree of vasogenic brain edema and vasculitic phenomena increasing the rate of neurological sequelae [17]. According to our case, we suggest than the neurophysiologic expression of these pathological data may present as PLEDs which could be considered as a manifestation of an increased neuronal damage.

## Competing interests

The authors declare that they have no competing interests.

## Authors' contributions

FHF, EFD, TS and JGG were involved in the direct care of the patient; JP performed the EEG. In addition FHF and JGG were responsible for drafting the manuscript. All author have read and approved the final version manuscript.
